# Narratives on why pregnant women delay seeking maternal health care during delivery and obstetric complications in rural Ghana

**DOI:** 10.1186/s12884-019-2414-4

**Published:** 2019-07-23

**Authors:** Joshua Sumankuuro, Memuna Yankasa Mahama, Judith Crockett, Shaoyu Wang, Jeanine Young

**Affiliations:** 1 Youth Alive Ghana, P.O Box TL 1708, Tamale, Ghana; 2 School of Community Health, Faculty of Science, Charles Sturt University, Orange, NSW Australia; 3 Department of Health Education, School of Public Health, Kwame Nkrumah University of Science and Technology, Kumasi, Ghana; 4 School of Nursing, Midwifery and Paramedicine, University of the Sunshine Coast, Brisbane, Queensland Australia

**Keywords:** Maternal healthcare, Barriers, Cultural beliefs, Traditional norms, Ghana

## Abstract

**Background:**

Despite the many maternal healthcare policy programmes in Ghana such as free the antenatal care (ANC) and the fee-exemption policy under the National Health Insurance Scheme, among others, the country has yet to make substantial improvements in addressing low skilled care utilisation in pregnancy and delivery. From previous studies, maternal mortality has been linked to women’s healthcare decision-making power at the household level in many low and middle-income countries. Thus, a pregnant women’s ability to choose a healthcare provider, act on her preferences, and to be sufficiently financially empowered to take the lead in deciding on reproductive and pregnancy care has significant effects on service utilisation outcomes. Therefore, we explored rural community-level barriers to seeking care related to obstetric complications and delivery from the perspectives of mothers, youth, opinion leaders and healthcare providers in Nadowli-Kaleo and Daffiama-Bussie-Issa districts in the Upper West Region of Ghana.

**Methods:**

This exploratory qualitative study was based on the narratives of women, health providers and community stakeholders regarding the expectant women’s autonomy to decide and utilise maternal care. To achieve maximal diversity of responses, purposive sampling procedures were followed in selecting 16 health professionals, three traditional birth attendants and 240 community members (opinion leaders, youth and non-pregnant women) who participated in individual depth interviews and focus group discussions.

**Results:**

Women’s lack of autonomy to seek care without prior permission, perceived quality care of traditional birth attendants, stigmatisation of unplanned pregnancies and cultural beliefs associated with late disclosure of childbirth labour all delayed mothers timely use of skilled care in the study communities. These barriers compounded problems arising from communities that are geographically isolated from hospital care.

**Conclusions:**

Decisions about seeking maternal care were usually made by the expectant woman’s husband and family without providing adequate support to pregnant women during the latter stages of pregnancy and delivery. We conclude that this is primarily a cultural issue**.** The study recommends a change in the approach to community-level health education campaigns for maximum impacts through the increased involvement of men and families in health service delivery and utilisation.

**Electronic supplementary material:**

The online version of this article (10.1186/s12884-019-2414-4) contains supplementary material, which is available to authorized users.

## Background

Skilled attendance during birth contributes significantly to positive delivery outcomes, with estimated maternal deaths between 1990 and 2015 reducing from 543,000 to 239,000 per 100,000 live births in low and middle-income countries compared to an estimated less than 12 per 100,000 women giving birth by skilled intervention in high-income countries such as Australia [1, 2]. Globally, 71% of births were assisted by skilled health professionals in 2014, which was an increase from 59% in 1990. Also, in sub-Saharan Africa and South Asia, an estimated 68% of pregnant women in these regions receive skilled health care during delivery [1]. From the low use of skilled attendance at birth (SBAs), an estimated 88% of all maternal deaths occur in sub-Saharan Africa and Southern Asia [1].

The Ghana Maternal Health Survey (GMHS) of key indicators found that 350 maternal deaths per 100,000 live births occurred in only 2017 [3]. About 75% of these deaths are caused by postpartum haemorrhage, infections, pre-eclampsia and eclampsia, birth complications and unsafe termination of pregnancy. Another 19% of mothers experienced complications during delivery, and commonly cited problems were vaginal bleeding, oedema/pre-eclampsia, blurry vision and prolonged labour [3, 4]. All these causes of deaths and complications are avoidable. From the literature, pregnant women in rural Ghana are at extreme risks of obstetric complications and have less access to hospital care than those in urban communities [3, 5]. Addressing these obstetric problems requires skilled attention. Although about 89% of pregnant women who participated in the 2017 GMHS had completed at least four antenatal visits and institutional deliveries were encouraged, 68% of births in rural communities in Ghana occurred without skilled attendance [3].

Maternal health policy initiatives such as free antenatal care (ANC) introduced in 2008 and the fee-exemption policy of Ghana’s health insurance scheme, for delivery care in 2005, among others, aimed to reduce health access barriers. However, in reality, Ghana has yet to make substantial improvements in addressing low skilled care utilisation in pregnancy and delivery. Thus, in the literature, maternal mortality has been linked to women’s healthcare decision-making power at the household level in many low and middle-income countries [6–13]. In some parts of Asia, distance to the health facilities, cost of access to skilled care, practices at health facilities such as horizontal birth position, episiotomies, the presence of male staff and mothers’ desire to have family members nearby were common reasons for home deliveries [14]. Also, in some communities in Ghana and other sub-Saharan African countries, decisions regarding birth location were commonly made by the woman’s husband, mother, mother-in-law or other relatives [15–17]. Thus, a pregnant women’s ability to choose a healthcare provider, act on her preferences, and to be sufficiently financially empowered to take the lead in deciding on reproductive and pregnancy care has significant impacts on service utilisation outcomes [6–9].

Different reasons account for deliveries outside health facility and non-use of skilled care during obstetric complications. Factors impacting timely utilisation and delivery of appropriate care during obstetric problems were categorised into three delays by Thaddeus and Maine [9]. These three delays – seeking care at the onset of labour (I), identifying and reaching a health facility (II) and receiving quality and timely care on arrival at the health facility (III), have had a significant negative impact on numerous maternal health interventions to date. The barriers to reaching the health facility (delay II) and receiving timely quality maternal care (delay III) were most commonly cited in the literature as distance, bad road networks, scarce vehicular transport services, inadequate referral management during complications, low family income and the breakdown in the health system [3, 5, 8, 10]. More recent studies show that inadequate autonomy, maternal support and cultural barriers to utilising timely and appropriate maternal care have also had a significant impact on service use and is associated with maternal health outcomes [11–14]. For example, family support during prenatal and delivery care was reported as a positive antecedent to mothers’ willingness and ability to utilise timely care at a health facility [4, 7, 13]. Issues of sociocultural beliefs and practices impacting on maternal freedom are widely reported in Ghana and other low and middle-income settings [6, 15]; it is well-known in the rural communities of many societies, men make decisions for women in most aspects of their lives [8, 9, 14]. Although some theorists view this as the subjugation of women’s freedom to men, others believe it is a way of preserving cultural heritage [16]. Some women believe men making decisions were helpful, while others express a contrary viewpoint [7, 9, 17]. Overall, lack of support, traditional norms and limited freedom of pregnant women in many low-income communities are negatively associated with women’s ability to utilise appropriate and timely care, with profound implications for the efforts of caregivers.

Therefore, this study addresses the question: what are the rural community-level barriers to seeking care related to obstetric complications and delivery from the perspectives of mothers, youth, opinion leaders and healthcare providers in Nadowli-Kaleo and Daffiama-Bussie-Issa districts in Ghana?

We explore this in the practical context of delay I of Thaddeus and Maine [9] and then suggest potential interventions for addressing the challenges identified.

## Material and methods

### Research setting

Nadowli-Kaleo district (NKD) and Daffiama-Bussie-Issa district (DBID) in the Upper West Region of Ghana are the study locations. According to the 2010 Population and Housing Census in Ghana, the two districts have a population of 61561 and 32827 respectively [18, 19], which account for 13.5% of the region’s population. The female to male ratio in DBID is 94.7 and 87.6 in NKD [18, 19]. However, there were more literate males than females aged 11 years old and more; 53.0% versus 47.0% in NKD and 48.2% versus 37.0% for DBID. The majority of the people are youth, and the proportion of males to females with secondary level education or higher was respectively 5 and 2.8%. Overall, six out of ten people in both districts could read and write a language [18, 19].

Economically, the districts are very deprived and dominated by subsistence farming, with more than 80% of the population not having formal sector employment [18, 19]. Subsistence farming accounts for over 70% of the labour force, and sorghum, millet, maize, groundnut and beans are the leading food crops grown. Also, nearly all families engaged in animal rearing and poultry farming with cattle, goats, sheep, pigs and poultry as the most common ones in the area [18, 19]. Women generally have little influence in decision-making pertaining to their health in these communities, particularly those who are uneducated and less educated [7, 12, 20–33]. There were cases of teenage pregnancies, which contributed to poor pregnancy outcomes and obstetric complications in most pockets of these districts [4].

#### Study design

This was a qualitative exploratory study that the authors conducted over 10 months from February to May 2016 and from January to May 2017.

### Community selection

The study was conducted in eight communities in the two districts. The two districts in 2015 were made up of 16 sub-district health structures [Health Centres and community-based health and planning service (CHPS) compound or clinic], five in Daffiama-Bussie-Issa district and 11 in Nadowli-Kaleo district. Purposive sampling criteria was used in selecting communities within the districts to maximise the diversity of experiences. First, eight sub-districts were chosen at random from the list of all 16 sub-districts. From the eight, we identified communities with a Health Centre and those without a Health Centre or with CHPS compounds/clinics. At this stage, one community from each sub-district was purposively selected, with equal consideration for those served by a Health Centre facility and those with a CHPS facility or clinic. This procedure led to the selection of eight study communities – four in each study district.

### Research participants

A total of 259 participants (240 FGD participants, 16 healthcare providers and 3 traditional birth attendants) took part in the research. Focus groups comprised non-pregnant women who had childbirth experiences, youth leaders (18–35 years of age) and community opinion leaders. The ages of participants ranged from 20 to 69 years. Apart from the healthcare providers, most of the participants had no formal education.

### Participant recruitment and selection

From the eight study communities, we used purposive sampling technique to choose participants who were core members of community development and maternal care programmes in the various community levels [12, 33]. Purposive sampling was used to help select stakeholders in maternal and newborn health, that will provide relevant information to address the research aims.

For the focus group cohort, the research team worked with local community leaders who gave permission to access their communities and served as informants and helped in recruiting research participants [33]. Two key recruitment techniques were adopted to recruit research participants. First, the research team repeatedly advertised study aims during the village market days using the “town criers” combined with “standing on strategic corners” of the communities. The research team carried out participant selection. A pre-determined inclusion criteria of participants’ willingness and availability, and being able to consent to participation personally, was used in composing the groups. The participants were approached directly by the research team, who explained the rationale of the study, established their eligibility and obtained their voluntary consent before they were included in focus groups. Twenty-four (24) units of FGDs and 22 individual depth interviews were completed. For the composition and participants, three focus groups of ten members in each group, were constituted for each community: a women’s group (*n* = 80), a youth group (*n* = 80; men = 40, women = 40), and opinion leaders’ group (*n* = 80; men = 58, women = 22). Therefore, a total of twenty-four (24) units of FGDs were conducted. The groups’ composition were partly informed by the literature and the need to obtain women’s experiences by providing them with the freedom to express their views in women’s only groups.

For the healthcare staff cohort, recruitment through “door knocking” was complemented with the provision of information sheets highlighting the study objectives randomly distributed in the various health facilities. Sixteen (16) healthcare professionals working at the Ghana Health Service and involved in maternal service delivery also participated, comprising a medical director, pharmacist, head of the maternity department of the hospital, and eight heads (midwives) of sub-district health facilities (health centres and community-based health and planning service compounds) from the eight research sites. Five were professional midwives, while the other three were community health nurses/enrolled nurses. Five other nurses (three nurses plus the two district directors of health services), who were not directly involved in antenatal care services delivery were purposively selected to provide information on their general experiences of maternal and newborn healthcare utilisation.

Three traditional birth attendants serving the study communities but residing outside them were also contacted, and those who gave their voluntary consent were included in the research.

### Research instruments

An open-ended/semi-structured question guide was used in collecting data for the study. The research questions were designed to gather answers related to family and community support to mothers, pregnant women’s freedom to decide on healthcare utilisation, the level of family support provided to the pregnant woman and barriers to access and utilise timely and skilled maternal care. Discussions also explored women’s freedom to use family resources during pregnancy and labour without fear or intimidation and the implications of these factors on skilled maternal care utilisation (see Additional file 1).

### Data collection

Individual depth interviews (IDIs) and focus group discussions (FGDs) were the techniques used to collect data from healthcare providers/traditional birth attendants and community members, respectively. JS and MYM received substantial training in different data collection techniques before the study, and we collected all the data, in collaboration with JC, SW and JY. The multiple sources of data using different techniques allowed for triangulation of the perspectives. Audio-tape recordings were clustered responses/quotes from FGD units. Each interview was carried out through face-to-face interaction, providing the researcher with the opportunity to clarify vague or unclear responses, and to pick up on social cues. Interviews with health professionals were completed in English while focus group discussions were conducted in the local language (“Dagaare”). FGDs sessions were held for the separate categories of participants to avoid intimidation and cultural nuances that may impact on discussants’ freedom to express opinions concerning the culture and household set-up in the Upper West Region of Ghana. The results of the focus group sessions and individual depth interviews pertaining to the research question are reported in this paper.

#### Quality control

The team had a minimum of a postgraduate degree in public health, and two researchers (JS and MYM) are proficient in the local language, “Dagaare”. JS and MYM received extensive training on ethics in research, data integrity and confidentiality issues of participants and had a firm grounding in interview questioning and data management before the study. All authors are experts in qualitative research. The research team were in regular communication to ensure the research was conducted as designed. Play-back approach of audio recordings to interview and focus group participants was used to confirm their submissions before leaving the research community. Reviews of any adverse impacts such as participant recruitment and selection criteria and those that declined were noted, discussed and adjusted during the study. During the data collection, the research team ensured data relevance and integrity processes were observed. All data were securely stored in a password-protected computer to ensure data privacy. Data processing and analysis involved prolonged engagement with the data by all authors. Reliability of the findings was assessed by two external experts who are familiar with qualitative research.

### Analysis

After the data collection, audio recordings of the FGDs were transcribed verbatim in *Dagaare* and translated to English and validated by language specialists from the Ghana Insitute of Languages to ensure rigour. However, interviews with healthcare providers were transcribed directly in English. The hand-written transcripts were typed and imported into NVivo version 11. Transcripts were studied to identify patterns which emerged as broad topics. Content analysis was done electronically and manually to index themes and factors and direct quotes selected to illustrate the factors and broader themes. Processing of each dataset was handled separately. Patterns within the responses in the transcripts was analysed deductively by coding concepts and main ideas. The research team conducted the coding independently and reconciled any differences that emerged. Data analysis was an iterative process and coding was carried out individually among all researchers, and any differences reconciled appropriately.

#### Ethics consideration

The study received ethics approval from Charles Sturt Human Research Ethics Committee [Protocol numbers: H16013 and H16178] and Navrongo Health Research Centre [Protocol number: NHRCIRB345]. The Regional Health Directorate of Upper West Region and the two districts also gave written support for the conduct of the study. Written informed consent (signed or thumbprint) was obtained from all participants, and participation was entirely voluntary.

## Results

Three themes and nine factors emerged deductively from the electronic search and manual coding and analysis of the transcripts and field notes. The themes include a) community cultural influence on maternal care utilisation b) social behaviours impacting maternal support and c) community-level interventions to improve on skilled care utilisation (Table 1).

**Table 1:** Themes and factors

Themes	Factors
Community cultural influence on maternal care utilisation	1. *Low maternal freedom* 2. *Late disclosure of labour* 3. *Perceive better care from traditional birth attendants* 4. *Stigmatisation of unplanned pregnancies*
Social behaviours impacting maternal support	5. *Unsupportive attitudes of spouses during pregnancy* 6. *Perceived harmful community beliefs against supporting mothers during labour* 7. *Negative attitudes of expectant mothers and nurses*
Community-level interventions to improve on skilled care utilisation	8. *Intensify community/sectional education programme* 9. *Encourage men involvement in maternal service uptake*

### Community cultural influence on maternal care utilisation

#### Low maternal freedom

Skilled care during pregnancy and birth are encouraged to reduce avoidable maternal and newborn morbidities and mortalities. To achieve this, expectant mothers should take the lead in decisions about their health care utilisation. However, some women delayed starting antenatal care or seeking timely care during obstetric complications because they waited for permission from their husband or another family member before they could seek medical attention:

*Women do not have a say in these communities. If it is the antenatal care or even the labour, she is coming, or there is something like go to Nadowli… like referred her during labour or complications, it is the men that will take the decision. If even the woman has the money and willing to go, but who is she to say, oh take me and go! They will call her a strong woman, or you are now taking the place of men, I think the traditions are also affecting healthcare seeking [IDIs, Midwife, Nadowli-Kaleo District (NKD)].*

#### Late disclosure of labour

In the FGDs, the majority of women concorded that early disclosure of childbirth labour to the closest person at the time would prolong the progress of labour. Therefore, hiding labour was perceived as a way of ensuring fruitful and safe birth, although such practices had adverse impacts on the birth outcomes and often led to home delivery:

*Some refuse to give birth at the clinic because they believe, when labour is announced prematurely, it prolongs. So, they keep it indoors until they give birth. Some shout for assistance when the baby is almost out…, We keep telling them that water infusions and blood transfusions cannot be done during home births, and even if they become pale unless, at Nadowli hospital, the clinic does not transfuse blood [IDIs, Midwife, Daffiama-Bussie-Issa District (DBID)].*

Another healthcare provider agreed, saying:

*They try to hide the labour signs until it is severe, and they will now say I’m suffering; let’s go to the clinic. Some expectant mothers, if they are not fortunate to get here early, they will either give birth in the house or even on the way, which is pathetic. “Born before arrival”, they [expectant mothers] usually think they will reach the facility before the baby is delivered. They will be on the way to a health facility while the baby is coming and can’t be stopped, even on the road [IDIs, Midwife, NKD].*

This practice also prevents healthcare professionals from knowing about the onset of labour to help the mother. A nurse professional added:

*Some of the cultural traditions sometimes, especially labour that set in, they believe that it’s not good to report early to the health facility. If the woman reports early to the health facility, they think that it would delay the baby from coming early. The pregnant woman, therefore, will be there [at home], when they see the head out, you see them then trying to reach the health facility. Sometimes most of them even deliver on the way, or they called us to come and conduct delivery of the woman on the way, or the woman may reach the CHPS compound, and the baby is just getting out [IDIs, CHO, DBID].*

#### Perceive better care from traditional birth attendants

Despite the educational programmes by Ghana Health Service and the ban on TBA deliveries, there was continual patronage of their services:

*In a month, I do conduct five deliveries on average. During the last Ramadan fasting (in the year 2016), I had no chance to rest at home. I conducted deliveries throughout that period. Sometimes clients wished I could travel to villages to provide services such as attend to women in labour [IDIs, TBA3, DBID].*

Another TBA mentioned that she could conduct 15 deliveries on average per month and only referred complicated labour to the hospital:

*I could conduct about fifteen deliveries a month. They used to come for me even at night to different communities to go and attend to women in labour. Sometimes, in return, I could come to find another expectant mother waiting for me to supervise her delivery. During the farming season, for instance, I could go to the farm, and people will follow-up there for me to go home and conduct deliveries. I could attend to deliveries throughout the day and at night. When I was unable to conduct a delivery, I referred them to Nadowli hospital [IDIs, TBA1, NKD].*

The mothers perceived that traditional birth attendants (TBAs) provide the required care for their pregnancies and ensure comfort, which encouraged the continued patronage of their services:

*I can palpate it to the extent that even when it is breech, I can diagnose and reposition the baby to cephalic to make delivery easy [IDIs, TBA2, DBID].*

The perceived beliefs and trust in the TBAs’ care contributed to more home deliveries than initially anticipated.

#### Stigmatisation of unplanned pregnancies

The embarrassment from unplanned pregnancies caused late commencement of antenatal care (ANC), which was in turn related to low levels of family planning. In some communities, family planning was perceived as a violation of traditional values and women were prevented from using family planning (FP) services, particularly in Bussie, Woggu and Naro and Korinyiri communities. Women who had unplanned pregnancies felt embarrassed seeking early skilled attention during labour:

*Exactly, we have a problem with the ANC too. Let me even start with family planning. FP, they say if we are doing it well, most people will not get pregnant at the time they want. There are communities we know; they come just to tell us ‘we don’t allow our women to do FP. We have put a bye-law/curse on the women. Any woman that will come and do FP will die with it, or something will just happen, and the woman will go [die], and they will know that she has done FP’. It’s hell, and because of that, the women always hide and come and take the FP, but when the husband gets to know, then it becomes a problem [IDIs, Midwife, DBID].*

The FGDs with the opinion leaders also revealed the same belief pattern:

*For instance, my wife once secretly went for FP services without my consent. When I discovered it, I forced her to go and have it removed from the arm. She conceived after removing it and refused to go for ANC and gave birth at home. Some women hide from us (husbands). My wife did not inform me of going for it [FP services], which was the reason I forced the nurses to have it removed [a man, FGDs, opinion leaders, DBID].*

### Social behaviours impacting maternal support

#### Unsupportive attitudes of spouses during pregnancy

Pregnant women were not supported to reach a health facility, with families usually only getting involved when mothers were referred from the health centre or CHPS compound to a hospital. Some husbands declined to assist their spouses to reach a suitable health facility for laboratory tests and health insurance card renewals. This lack of support was at least partly related to the status of expectant mothers, and women more broadly, in the communities. Pregnancy was perceived as ‘women’s business’, and as a result, there was a stigma attached to men accompanying their wives to ANC. Men who accompanied their spouses were seen as being ‘charmed by the wife’:

*That some they tell them [husbands], and they [husbands] will, in turn, tell you [expectant mother] it is women’s matter. Women and madam’s [midwife] matter. Others too say they feel shy or other men will call them names…. you [the husbands] are a fool, your [husbands] woman has done some ‘juju’ [charm] on you to be following her always, so that she will be going for ANC and you have to carry her handbag and follow her to clinic [IDIs, Midwife, NKD].*

Not only did men attach a stigma to the father’s attendance at ANC, but there is also stigma attached by expectant mothers to the participation of fathers in ANC, predominantly over concerns that other attendees would know who the baby’s father is:

*It’s a two-way thing. Others do that. Other mothers do want, but the men refuse to go for ANC. When we do ask them, they laugh? They say the men won’t come [IDIs, Midwife, DBID].*

A similar observation was found during the FGDs:

*Some men who appear rugged have been prevented by their wives from accompanying them to the health facility, that she would feel embarrassed when her colleague pregnant women get to know that the rugged man is her partner. They usually don’t want other women to recognise that a particular man is responsible for her pregnancy [FGDs, Opinion leaders, NKD].*

In some families, accompanying the expectant mother to seek care was not considered necessary; it was more important that they be able to maintain their economic responsibilities:

*They won’t waste their time coming down here for whatever. Some too that the men will say they are going to their farms. Hence, they leave the mothers to seek ANC, and they will also go to their farms so that they can do something [IDIs, Midwife, DBID].*

#### Perceived harmful community beliefs against supporting mothers during labour

Although spousal involvement during pregnancy and birth are encouraged, there were traditions in some communities barring men from supporting mothers during labour, particularly about concerns about the man seeing the newborn. Men who adhere to the “can’t see neonate” belief system were prohibited from supporting the mother during the first month immediately after delivery. These traditions were inherited from their ancestors, who believed it was the responsibility of the gods (not man) to protect the infant.

*It is also believed men must not see a neonate who is still less than three weeks old (for boys) and four weeks old (for girls). Families who have joined such belief system prohibit it. However, men who have not been initiated into the belief system are free to see the neonate. Therefore, when the husband wants to converse with the wife [mother in early postpartum], she has to leave the baby in her room and go out to meet him. Alternatively, she can cover the whole body of the baby so that the man does not see the naked body. A break of the belief subjects the baby to perpetual and chronic sickness if not treated early; the newborn would die within a few days. The man who sees the neonate will also become blind until the gods are pacified for him to recover sight. Therefore, men are not involved during labour and the early postpartum due to this belief [FGDs, youth leaders, NKD].*

Not unlike the men, the adult women had their belief system, which prevents them from seeing the neonate of their peers. Supposedly, the newborn could die if the child’s mother was not a member of the said tradition. The conditions about the belief include all forms of supporting an expectant mother in labour or a newly delivered mother who may need help from other pregnant and nonpregnant women. Some expectant mothers do not want to have a birth in a health facility because of beliefs associated with other women of a different belief system seeing newborns. This was common in Siruu and Charikpong enclave where women who see or helped mothers to draw water or carry headload or give support in the facility/ward were seen as likely to ‘infest’ their newborn.

*The women also have “bowl” and “calabash” beliefs. Usually, they are initiated into these for the safety of the pregnancy and protection over the neonate. Hence, women who are into the belief system are not allowed to see a neonate who is less than a month or even support expectant mother carry headload. The effects are that the expectant mother who does not form part of the belief system could have a stillbirth or the newborn could fall sick, and if not detected early, the baby might die [FGDs, youth leaders, NKD].*

#### Negative attitudes of expectant mothers and nurses

The spouses reported women as having a negative attitude towards their husband’s involvement, such as helping her reach a health facility during labour:

*The women have some negative attitude(s). Often, when men suspect they are due and probably inquiring, they refuse to declare the progress of the pregnancy. When the husband takes off to farm or elsewhere, the expectant mother will then begin to look around for motorist to take her to the clinic. Such delays lead to childbirths on the wayside. The clinic is within the community; but we have had so many wayside births [a man, FGDs, opinion leaders, DBID].*

Negative attitudes of nurses towards pregnant women, including those in labour, also detracted from their ability to receive care in an emergency:

*When we give them a knock on their doors to attend to an emergency at night, they refuse to provide care, not even a pain reliever. When the client family insist, they just write a referral letter for you [FGDs, non-pregnant women, NKD].*

### Community-level interventions to improve on skilled care utilisation

The study participants identified possible interventions to address the barriers to health care utilisation during obstetric problem and delivery in the area.

#### Intensify community/sectional education programmes

The low awareness of basic obstetric histories and the progress of the pregnancy, coupled with birth unpreparedness and not being ready for complications, gave rise to a request for increased birth preparedness and complication readiness education during ANC:

*We need more awareness creation among expectant mothers to know the significance of ANC, most especially among the young ladies and primis, so they can begin ANC early and continue through to childbirth. This will ensure safe childbirth unto a healthy baby [FDGs, non-pregnant women, DBID].*

#### Encourage men involvement in maternal service uptake

There are also benefits to be derived from providing maternal education related to spousal participation in maternity care, including the potential to increase the willingness and freedom of pregnant women to participate in and comply with directives during obstetric and newborn referrals. The advantages of increased involvement by men are illustrated by a participant at Siruu community who regularly supports his wife financially and with a motorbike to reach Nadowli hospital for laboratory investigations and during obstetric referral, to ensure her safety and timely access to appropriate care, as iterated:

*One support some men can give is the peace of mind for her to go through the gestation period successfully. Anytime my wife conceived, I pick her with my motorbike to receive laboratory tests at Nadowli so that she can commence ANC actual on time. I also ensure her NHIS card is active. I have taken her to ANC before in her recent conception. We also support them by reducing the volume of work whenever we go to the farm. She is allowed to do minimal activities [a man, FGDs, youth leaders, NKD].*

While it is generally considered culturally inappropriate for men to seek care with their spouses, it was reported in the interviews that some younger men were receptive to helping and accompanying their spouses to health facilities:

*Some men are trying. They go with the wives after the education whatever you [midwife/nurse] need to tell them we [midwife/nurse] tell the husband. However, most of them don’t bring their spouse. Yes, the men do not participate in health activities. When we organise the durbars (mass community gathering for health promotion campaigns, festivities, etc), just a few come, even though we encourage them. Just a few follow their wives to come [IDIs, Midwife, NKD].*

## Discussion

Providing skilled care faces significant challenges at the community/village level in Ghana. While there are substantial benefits of early decisions to seek attention during complications and labour as it may help reduce the time and difficulty reaching and receiving appropriate and quality care, barriers to timely access were found as crucial issues in maternal care provision in this study. The barriers were associated with community cultural beliefs, which influenced the traditional household/family set-up. The results of this cultural heritage led to the late disclosure of labour, limited the expectant mother’s autonomy to lead in care-seeking decisions, beliefs that prevent community members from supporting expectant mothers in childbearing roles, and the increased attendance of births by traditional birth attendants. Overall, these barriers contributed to home deliveries and impacted healthcare providers’ ability to help women achieve skilled attention for obstetric problems, childbirth and general reproductive health.

Using participants’ narratives, two themes and ten factors were identified as critical barriers to care-seeking decisions during obstetric complications and birth in rural settings about the delay I (deciding to seek care). Data was also collected regarding ways to address the identified barriers.

Maternal freedom in the context of maternal health means that the expectant mother leads in decisions in line with how she wants her pregnancy and healthy life to progress [15, 20]. Although women’s lack of freedom to decide and utilise timely care when they most need it was reported in several studies, the intensity of maternal health service programmes by the Ghana Health Service and the media networks ought to have reduced this pattern. However, this has not occurred, at least in many of the study communities where the traditional power structures within families and communities and the lower social position of women exist. Thus, many still have to obtain permission before seeking care.

These findings are consistent with recent reports from the Upper West Region [16], where, at the family level, women obeyed spouses fully, and most could not travel or seek healthcare without prior permission from the spouse or family head [7, 13]. This finding is in accord with previous studies in the region and similar low and middle-income countries [7, 13, 21]. Although significant disparities existed between educated and uneducated women in the very remote areas compared to peri-urban and urban settings, male dominance in decisions concerning health service use continued to have negative impacts on pregnancy outcomes and contributed to obstetric fistulas in some rural settings in Ghana and Ethiopia [4, 22]. These poor outcomes occur regardless of the number of contacts a woman may make with the health system during the pregnancy. For example, Bayu et al. [23] in a community-based follow-up quantitative study in Ethiopia found that mothers who received timely permission to seek care and were supported by their partners were more likely to give birth at a health facility. Less autonomous mothers were indecisive in choosing the place of delivery. Bhatta [24] also noted that the involvement of men at Kathmandu, Nepal, in prenatal care could encourage them to provide support in preparing expectant mothers for obstetric complications.

Similarly, Gabrysch et al. [6] in a nationwide study using the national demographic and survey data in Zambia reported that women’s freedom was positively associated with access to a health facility during delivery. They found that educated mothers were more likely to have some degree of autonomy and would prefer health facility delivery with access to skilled maternity care. Also, a systematic review in sub-Saharan Africa reported extensively on the negative influence of maternal support and freedom from family and community as a significant determinant of health facility delivery [25]. For example, expectant mothers were required to obtain permission from spouses, in-laws and family heads to seek facility care in a qualitative study in the northern region of Ghana [26]. The outcome of these controls were avoidable home deliveries [26]. As was the case in the study areas, husbands/partners decided on ANC attendance in Bangladesh and Nepal, and most expectant mothers without the freedom were less likely to complete four ANC visits [27, 28]. These studies involved women from both rural and urban communities, although joint decision making on maternal healthcare was lower (about 12%) among the rural residents. Other research reported that women could commence ANC early and complete more than four visits yet give birth without skilled supervision due to lack of autonomy among the Massais in Northern Tanzania [29].

A variety of reasons exist for the lack of freedom among individual mothers in this study. First, some women were prevented from unauthorised movements due to fears that they may use family planning without the man’s knowledge. The lack of spousal approval was often related to fear of family planning and expectations that women work rather than receive professional health care. The finding is consistent with previous studies in the Upper West and Northern Regions, which observed that controls over women were associated with preservation of religious and sociocultural beliefs and practices/heritage [30]. They found that threats of sociocultural beliefs were used to control and discourage mothers from using family planning services, and opposition from husbands was a primary reason for non-use of FP services [30]. The placing of curses on mothers reported in similar study communities was confirmed in this study as a critical barrier for pregnant women to utilise health facility care, even in labour genuinely and or when they experienced complications. Engaging mothers as farm hands was preferred to honouring health appointments during the farming season [13], and similar reasons were reported in Ethiopia for mothers who missed out on their plans to give birth at a health care setting [23].

Overall, the woman’s lower social position and influence of cultural beliefs and practices may significantly affect her ability to make early decisions on how she will reach the healthcare setting during labour or if she experienced obstetric complications. Although the non-supportive attitude of men towards mothers occurred within home-settings, it was fuelled by sociocultural behaviours towards women’s use of reproductive health services and traditional beliefs associated with “can’t see the neonate” which put a substantial burden on women in childbearing roles. These are all forms of maltreatment to mothers, and the consequences were women having little hope and courage in using skilled delivery care for fear of the associated family traditions which contribute to home deliveries. Lack of spousal and family support during labour, which often reflected long-held cultural values, beliefs and attitudes also contributed to delays in seeking care during childbirth.

Concerning the views of the community members and healthcare providers, it was found that men did not give relevant logistical support to expectant mothers. This is a problem, given the geographical isolation, few access roads and scarce vehicular transport services available for a woman during labour or who are experiencing an emergency. Ambulatory services by the Ghana Ambulance Service exist in many towns and district capitals in Ghana, but there are fees which makes their services unaffordable aside from the lack of access to many rural communities [5, 8]. Women in this study area did not own motorbikes, although that was the dominant means of transport when seeking care [5, 10, 13]. It was found that men often did not accompany mothers during labour, although they may assist in emergencies. This finding is not new in the Upper West Region; in a study into women’s autonomy and family decision-making on access to healthcare Ganle et al. [16] found that family influence limited mother’s ability to utilise timely care.

Other significant findings in this study were late disclosure of childbirth labour, the stigma on unplanned pregnancies and the perceived efficacy of traditional birth attendants’ delivery care due to cultural beliefs. Delayed disclosure of labour may contribute to unskilled assistance (such as relatives and traditional birth attendants) and is premised on the belief that women who inform the closest person immediately can delay the delivery. This, in turn, results in fear among mothers. Even nurses/midwives were prevented from knowing about the onset of labour while they are at the health facility, often until labour progresses to the third stage. This was reported as a significant determinant of home and roadside deliveries and subsequent risk of puerperal and neonatal sepsis. This finding corroborates previous studies in other low and middle-income settings [21]. In a qualitative evaluation of causes of maternal deaths in rural central India, Jat et al. [20] reported similar beliefs contributing to late reporting to a health facility during labour. Similarly, a qualitative study in Lao People’s Democratic Republic found that factors such as the cost of services and negative treatments discouraged women from choosing the health facility delivery; the perceived effects of disclosing premature labour pains which may prolong labour largely contributed to late reporting to a health facility during labour [14].

From the FGDs, it was found that expectant mothers were often treated negatively by health professionals when they seek care from the primary health care facilities (health centres and CHPS compounds). The undignified treatments and failure of staff to recognise the urgency of the mothers’ health problem were found as influencing future decisions to use skilled care. This finding is consistent with those of previous studies in Ghana. In a qualitative assessment involving disaggregated participants on healthcare workers’ behaviours towards mothers during obstetric complications and labour in Ghana, it reported that mothers were verbally abused and suffered different forms of discrimination during care, which was positively associated with their choice of home and TBAs deliveries and the use of traditional healers during complications [26]. Similar findings were reported in the study districts [13]. Although Sumankuuro and colleagues found that stress from workloads was the cause of the negative attitudes towards mothers, it may not be ethical in many ways [13], as their [mothers’] ability to judge and appreciate staff loads in rural Ghana may be poor [17], given the low formal educational levels of women in general in the study area and our study participants [13, 18, 19]. Maltreatment of mothers when they seek primary health care was a determinant of health facility care during obstetric problems and delivery.

Overall, in Ghana, the introduction of decentralised maternal care at village-level, coupled with the fee-exemption policy for delivery services was meant to reduce distance and cost barriers to skilled care. However, cultural beliefs, as reported in this study and previous research in rural northern Ghana may thwart government efforts at achieving skilled supervision during childbirth.

### Implications for policy and practice

The roles men play in maternal care is reflected in the WHO ‘Protocol on focused antenatal care for low and middle-income countries’, with emphasis on active involvement of husbands/partners as an enabling factor for obtaining more significant net impact on prenatal care, rather than providing health messages and education to only expectant mothers [31, 32]. This is particularly important given that men often control the material resources of spouses including decision-making concerning the choice of the health service provider and time of use, the daily family activities and family size in most families in northern Ghana. From the findings, it would be an obvious decision by Ghana’s Health Ministry to adopt different strategies to addressing cultural influence, with focus on educating both men and women on the risks associated with delays deciding to seek to care during obstetric problems and labour [14]. Over the past decade, there have been a series of interventions and approaches implemented by Ghana’s Health Ministry and other partner organisations including regular community health education and promotion programmes using community-based health surveillance volunteers to assist in the campaigns and conduct of national immunisations exercises. Recently, mass community forums called *durbars* were a widely used approach. However, the interviews with healthcare providers revealed low attendance by men (who are mostly the primary target of health messages). Therefore, we suggest a new approach using existing separate sectional meetings/groups, which range from micro-loans groups to communal labour/support ones to convey health messages. These recommended smaller groups could be used for a piloted phase focusing on harmful community beliefs and practices. On the “can’t see neonate belief” system and mothers having to obtain permission to seek care, we suggest community-level engagement and dialoguing-approach with community elders, landowners and “belief” owners and other influential persons in the community be used [4, 14, 17]. The engagement will potentially unearth their intentions, which may lead to some consensus without eroding the cultural heritage.

Despite the many barriers to women’s healthcare-seeking decision-making, several participants suggested ways to address the situation. One strategy is to modify community-level health education campaigns targeting elders to help reduce the relevance attached to harmful beliefs and practices that impact on women’s health [14, 17]. This could have a more significant impact at eradicating these beliefs than the ‘traditional durbars’ which are rarely attended. It was also found that some men, particularly the younger ones, were receptive to maternal care and supported their spouses in pregnancy and during ill-health, which may motivate other men to join. Thus, engaging the communities, especially men in those communities, in behaviour-change communication to understand health risks and the need for their active participation in care-seeking may encourage them to embrace and support women and give women the freedom to seek care use family financial resources in pregnancy and delivery [8, 11]. This is particularly pertinent, knowing that men to make decisions for mothers in rural northern Ghana concerning reproductive health and childbearing roles; increasing active spousal involvement could relieve women of the burden of getting timely permission to seek appropriate and timely care at health facilities.

### Strengths and limitations

Although the literature provides sufficient evidence to support the use of the qualitative approach to obtaining an in-depth understanding of barriers to care during delivery and obstetric emergencies, the method does not reflect the reality, partly due to the fact that a purposive sampling procedure was followed in selecting the majority (all FGD participants and health professionals/managerial staff) of the participants, which could have its own biases. The study was not conducted in all communities in the research area, and it must be acknowledged that although these findings may be transferable, different barriers and determinants may exist in the other communities not sampled. For this reason, further studies are recommended in those areas to inform health decision-making policy in those specific communities. However, these limitations are unlikely to change the fundamental findings of this study.

The data processing and presentation of results were carried out using computer software (NVivo) which could have programming limitations to the user, and that could affect the analysis; thus, we augmented the computerised procedure with manual coding.

Also, the study mainly reported on participants’ opinions, and it is assumed that personal views are subjective. The interviews with healthcare providers and the FGD sessions were also conducted in the local language (“Dagaare”) and transcribed and translated into English by the researcher; although Dagaare is the mother tongue of the researcher, the processing stages could have flaws regarding language appreciation and interpretation errors which may affect the original intent of the participants. However, the validation of the transcripts and by language experts means that possible errors were minimised in the results. Summarised themes were also checked by participants who were then used to reflect on the interpretation, thereby reduced researchers’ intuition and personal judgements of results.

There may be some concerns raised over the shared ethnic and cultural background of participants and the researcher, and the potential for this to bias results. The potential adverse effects of the common background were addressed through proactive measures, including gathering data from multiple sources using multiple methods to facilitate triangulation. It helped to eliminate any potential biases in the results.

## Conclusion

This paper focused on rural community-level barriers to seeking care related to obstetric complications and delivery. We found that many of these barriers were associated with the beliefs and actions of men, particularly husbands, and other members of communities, especially older members. Seeking timely and appropriate maternity care with the full support of husbands/partners is more than a family issue; it is a cultural issue. National, regional and local initiatives are required to address each of these cultural barriers, with an emphasis on community sensitisation programs tailored to men and opinion leaders for maximum impact.

## Additional file

Additional file 1: Research instrument. (PDF 95 kb)

## Data Availability

The datasets used and analysed during the current study are available from the corresponding author on reasonable request.

## Abbreviations

ANC: Antenatal care
CHO: Community health officer
CHPS: Community-based health and planning services
DBID: Daffiama-Bussie-Issa District
FGDs: Focus group discussions
FP: Family planning
GMHS: Ghana Maternal Health Survey
GSS: Ghana Statistical Service
IDIs: Individual depth interviews
NKD: Nadowli-Kaleo district
TBA: Traditional birth attendant
WHO: World Health Organisation
