# Supplementary material for: Narratives on why pregnant women delay seeking maternal health care during delivery and obstetric complications in rural Ghana
Source: BMC Pregnancy Childbirth. 2019 Jul 23;19:260. doi: 10.1186/s12884-019-2414-4 (PMC6651920; doi:10.1186/s12884-019-2414-4)
Supplement: Supplementary file 1 — Research instrument. (PDF 95 kb) [file 12884_2019_2414_MOESM1_ESM.pdf]

Supplemental file: Research instrument

Manuscript title: Narratives on why pregnant women delay seeking maternal health care during delivery and obstetric complications in rural Ghana

Healthcare staff

1. In your opinion, what are the main causes of maternal deaths in the communities?
2. What are the main causes of neonatal deaths in the communities?
3. The first phase of the research showed that lack of maternal resources negatively impacts on care by midwives/nurses despite care being (technically) free to encourage ANC and skills attendance at birth. Why do you think this happens?
4. Research have shown that there are many social and cultural factors affecting birth preparedness and complication readiness interventions in the communities. What do you think are the most significant social and cultural influences maternal care in this community? Why do they have an impact?
5. What are the arrangements put in place to effect maternal referrals to next level of care?
6. In your view, in what ways does the fee-exemption policy of the national health insurance scheme (NHIS) affect maternal and newborn health care provision?
   a) Does it cover all aspects of maternal and newborn health?

7. What are the major issues pertaining in the skilled maternal service utilisation among expectant mothers and newborns?

8. In your opinion, what are the main causes of maternal deaths in the communities?

9. Research also showed that there are many social and cultural factors affecting birth preparedness and complication readiness interventions in the communities. What do you think are the most significant? For each factor, please explain why you think it is so significant.

11. a. what do you think stops women/expectant mothers from seeking skilled maternal care and on time?
12. b. What do you think stops women from wanting to give birth in a health facility?
13. What do you think are the best ways to prevent maternal deaths and still births in the community?
14. What are the factors preventing women/expectant mothers from seeking care early during pregnancy and childbirth in your community? Are there some cultural beliefs and practices affecting skilled maternal care uptake?
15. What will you suggest should be done to prevent pregnant women and new-borns from dying?

Participants: Traditional birth attendants in project sites

1. Do you still provide care during pregnancy and/or childbirth? If so, in what ways?
2. Approximately how many deliveries could you conduct in a year before the ban?

a) How many childbirths have you been involved in, on the average, in a year since the ban?

3. How has the ban affected expectant mothers' demand for your services?
4. Are there collaborations between you and the health facilities?
5. Are there some material and/or financial benefits you receive for:
   a) Providing prenatal care?
   b) Conducting childbirths?
6. Are there some peculiar reasons motivating your continued practice, despite the ban?
7. In your opinion, what are the causes of maternal deaths in the area?
8. In your opinion, what are the causes of neonatal deaths in the area?
9. What challenges do you face in your practice as a TBA?
10. What is your view on Ghana health service' ban on your services?
11. a. what do you think stops women from seeking skilled maternal care and on time?
12. b. What do you think stops women from wanting to give birth in a health facility?
13. c. What do you think are the best ways to prevent maternal deaths and still births in the community?
14. What are the factors preventing women/expectant mothers from seeking care early during pregnancy and childbirth in your community? Are there some cultural beliefs and practices affecting skilled maternal care uptake?
15. What will you suggest should be done to prevent pregnant women and new-borns from dying?

Focus group discussion guide

Participants: Opinion Leaders

1. What kinds of things/activities should a woman do when pregnant?

   - prompts – at home? – at work? – in the community

2. What do you think are the main causes of deaths of pregnant women or after delivery in this community/district?
3. What do you think are the main causes of deaths of newborns in this community/district?
4. What can you tell me about community initiatives towards ensuring safe motherhood?
5. What can you tell me about other settings (places) or groups where pregnancy related matters are talked about in the community?
6. What forms of support do these groupings offer to pregnant women?
7. What sorts of things does your family do (or do families do) to help ensure safe pregnancy and child birth?
8. What reproductive cultural practices exist in your communities?
9. a. what do you think stops women from seeking antenatal care?
b. What do you think stops women from wanting to give birth in a health facility?
10. What do you suggest are the roles of men in pregnancy?

11. Are men active as expected in supporting the woman during the period of pregnancy?
12. a. what do you think stops women from seeking skilled maternal care and on time?
. b. What do you think stops women from wanting to give birth in a health facility?
13. What do you think are the best ways to prevent maternal deaths and still births in the community?
14. What are the factors preventing women/expectant mothers from seeking care early during pregnancy and childbirth in your community? Are there some cultural beliefs and practices affecting skilled maternal care uptake?
15. What will you suggest should be done to prevent pregnant women and new-borns from dying?

Participants: Non-Pregnant Women
1. What are the causes of maternal deaths in your community?
2. What are the causes of Neonatal deaths in your community?
3. What things/activities should pregnant women do in your community?
5. If someone talked to you about birth preparedness and being ready for complications in pregnancy, what do you think they would be talking about?
6. What can you tell me about community initiatives towards ensuring safe motherhood?
7. What can you tell me about other settings (places) or groups where pregnancy related matters are talked about in the community?
8. What forms of support do these groupings offer to pregnant women?
9. What sorts of things does your family do (or do families do) to help ensure safe pregnancy and child birth?
10. What reproductive cultural practices exist in your communities?
11. a. what do you think stops women from seeking skilled maternal care and on time?
12. b. What do you think stops women from wanting to give birth in a health facility?
13. What do you think are the best ways to prevent maternal deaths and still births in the community?
14. What are the factors preventing women/expectant mothers from seeking care early during pregnancy and childbirth in your community? Are there some cultural beliefs and practices affecting skilled maternal care uptake?
15. What will you suggest should be done to prevent pregnant women and new-borns from dying?

Participants: Youth
1. If someone talked to you about birth preparedness and being prepared for complications in pregnancy, what do you think they would be talking about?
2. What are your perceptions of safe pregnancy?
3. What are the various kinds of support pregnant women receive from family members?
4. What community plans are there for emergency situations in pregnancy?
5. What reproductive cultural practices exist in the community?
6. How do you think these practices have some effects on pregnancy outcomes?
7. What are the factors preventing women/expectant mothers from seeking care early during pregnancy and childbirth in your community? Are there some cultural beliefs and practices affecting skilled maternal care uptake?
8. What do you think can be done in the community to improve the health of pregnant women and newborns?
